# Vitamin B6 alleviates osteoarthritis by suppressing inflammation and apoptosis

**DOI:** 10.1186/s12891-024-07530-x

**Published:** 2024-06-06

**Authors:** Zhaoyi Fang, Qingxiang Hu, Wenxin Liu

**Affiliations:** Department of Sports Medicine, National Center for Orthopaedics , Shanghai Jiao Tong University Affiliated Sixth People’s Hospital, No. 600 Yishan Road, Shanghai, 200233 China

**Keywords:** Osteoarthritis, Chondrocytes, Vitamin B6, Apoptosis, Cartilage, Interleukin-1β

## Abstract

**Background:**

Although various anti-inflammatory medicines are widely recommended for osteoarthritis (OA) treatment, no significantly clinical effect has been observed. This study aims to examine the effects of vitamin B6, a component that has been reported to be capable of alleviating inflammation and cell death in various diseases, on cartilage degeneration in OA.

**Methods:**

Collagen-induced arthritis (CIA) mice model were established and the severity of OA in cartilage was determined using the Osteoarthritis Research Society International (OARSI) scoring system. The mRNA and protein levels of indicators associated with extracellular matrix (ECM) metabolism, apoptosis and inflammation were detected. The effect of vitamin B6 (VB6) on the mice were assessed using HE staining and masson staining. The apoptosis rate of cells was assessed using TdT-mediated dUTP nick end labeling.

**Results:**

Our results showed a trend of improved OARSI score in mice treated with VB6, which remarkably inhibited the hyaline cartilage thickness, chondrocyte disordering, and knees hypertrophy. Moreover, the VB6 supplementation reduced the protein expression of pro-apoptosis indicators, including Bax and cleaved caspase-3 and raised the expression level of anti-apoptosis marker Bcl-2. Importantly, VB6 improved ECM metabolism in both in vivo and in vitro experiments.

**Conclusions:**

This study demonstrated that VB6 alleviates OA through regulating ECM metabolism, inflammation and apoptosis in chondrocytes and CIA mice. The findings in this study provide a theoretical basis for targeted therapy of OA, and further lay the theoretical foundation for studies of mechanisms of VB6 in treating OA.

**Supplementary Information:**

The online version contains supplementary material available at 10.1186/s12891-024-07530-x.

## Introduction

Osteoarthritis (OA), a degenerative joint dysfunction, is mainly characterized by joint pain and stiffness [1]. According to epidemiological statistics of OA, the incidence of OA in people over 65 years old can reach 50%, and the incidence of OA in people over 80 years old is as high as 80% [2]. Globally ranked among the leading causes of human disabilities, OA has gradually gained the attention of researchers since it highly impacts patient lives and its treatment usually requires colossal finance [3].

Articular joints are comprised of different tissues, including cartilage and bone, with distinctive structural and mechanical properties. OA is characterized first by a loss of cartilage which then, due to abnormal joint loading, leads to subchondral bone abnormalities driven by excessive bone remodeling [4]. The pathogenesis of OA is complex. Although several studies have previously identified some specific contributing factors, including pro-inflammatory factors such as tumor necrosis factor (TNF)-α and interleukin (IL)-1β [5, 6], which can stimulate the production of enzymes that break down cartilage and inhibit the production of protective molecules, leading to the development of OA [7], the mechanisms of OA onset and progression is not fully understood. The whole structure of cartilage components, including collagen, proteoglycans, and glycosaminoglycans, derives from a group of cells called chondrocytes. The self-repair process of chondrocytes is crucial in various pathological reactions; an extreme decrease of chondrocytes’ cell viability is associated with elevated chondrocytes apoptosis and perturbated homeostasis [8]. In addition, it was reported that suppressing chondrocytes apoptosis could help alleviating clinical symptoms and retarding OA pregression [9]. However, no significantly clinical effect on OA progression has been observed [10].

Previous studies have indicated that the concentration of vitamin B6 (VB6) is highly associated with pro-inflammatory components in cells [11]. The plasma pyridoxal 5-phosphate, an active biological form of vitamin B6, can regulate the flux of tumor necrosis factor-α (TNF-α) and other important inflammatory markers such as the C-reactive protein and erythrocyte sedimentation rate [12, 13]. In addition, the inhibition of vitamin B6 has been reported to induce OA symptoms like cartilage disorders [14]. A recent study suggested clinical practice concerning the beneficial utilization of vitamin B6 to block the inflammatory reactions in patients who have rheumatoid arthritis. The authors demonstrated that a 100 mg/day treatment of vitamin B6 decreased the pro-inflammatory level of cytokine in arthritis patients [15]. It is important to note that VB6 can reverse reactive oxygen species-induced adverse effects by inhibiting the xanthine oxidase activity [16], and the supplementation of vitamin B6 might protect the cartilage by modulating the ROS level in OA [17].

This study aims to investigate the role of vitamin B6 in anti-apoptotic in OA. And this study provides an experimental baseline regarding the clinical applications and medicinal effectiveness of vitamin B6 in the treatment of OA.

## Materials and methods

### Animals and induction of collagen-induced arthritis

The following experiment was conducted in accordance with ARRIVE guidelines and approved by the ethics committee of the Shanghai Jiao Tong University. Collagen-induced arthritis (CIA) model was established in mice. C57BL/6 mice (8 weeks) were obtained from Shanghai SLAC Laboratory Animal Co., Ltd and randomly separated into three groups: sham group, CIA group, and CIA + VB6 group (*n* = 12 per group). For CIA group, type II collagen (200 µg) dissolved in DMSO was added to an equal volume of Freund’s Complete Adjuvant and emulsified in the ice bath. Then, 100µL of the emulsified solution was administrated through intradermal injection at the base of the tail, and this immunization was boosted 3 weeks later. For the CIA + VB6 group, mice were treated with VB6 (40 mg/kg/day) once per day for 4 weeks before the CIA operation and the induction of the CIA was the same as the CIA group.

### Histological assessment of CIA

The hind limbs were fixed with 4% paraformaldehyde (Cat# G1101, Servicebio, China) and then decalcified using EDTA solution (Cat# G1105, Servicebio, China). Then, 5 μm thickness of tissues was cut from paraffin wax blocks, stained with Hematoxylin-eosin or masson staining [18], and the OARSI scoring system was monitored to measure the severity of OA [19].

### The isolation of articular cartilage

Under sterile conditions, the articular cartilage was extracted from the knees and femoral heads of C57BL/6 mice at 8 weeks of age. The well-preserved cartilages of mice were rinsed with PBS and then segmented. Each piece of cartilage was digested for 30 min in 0.25% Trypsin-EDTA solution and then in 0.2% collagenase type II in Dulbecco’s modified Eagle’s medium (DMEM) for 2 h at 37 °C. Therefore, the released chondrocytes were collected in DMEM/10% FBS solution. The chondrocytes were seeded in plates and then treated with 10 ng/ml of IL-1β based on the groups including control group, IL-1β group, IL-1β + 25µM vitamin B6 (VB6 25µM), and IL-1β + 100µM vitamin B6 (VB6 100µM) group.

### Cell viability

To evaluate the potential cytotoxicity of, the Cell counting kit-8 (CCK-8) analysis was carried out. The CCK-8 analysis was carried out according to the manufacturer instruction (Dojindo, Japan). Cells were rinsed and the CCK-8 solution was added into the plates. Absorbance density (OD) values at 480 nm measured through a microplate reader (Bio-Tek, USA) was used to evaluate the cell viability.

### Enzyme-linked immunosorbent assay

The cells were cultured in 96-well plates, and experiments were performed when the cell confluence reached 80-90%. The supernatants were obtained by centrifugation at 1000 g for 5 min and sterilized by filtration at 0.22 μm; then, IL-1β and IL-6 were measured following the manufacturer’s instruction of the ELISA kit.

### TdT-mediated dUTP nick end labeling (TUNEL) assay

The slides were dewaxed and hydrolyzed in xylene three times for 5 min, two-times 10 min in 100% ethanol, two-times 10 min 95% ethanol, and two-times 5 min ddH_2_O, respectively. Furthermore, the antigen retrieval was carried out in 20 min at 37 °C using Protein K solution (20 µg/mL) and washed slides with PBS in 5 replicates. Then, we used a fresh-made TUNEL solution for the incubation step in a humidified chamber (37 °C for 1 h). Finally, the slides were transferred on the DAPI (Sigma), five times cleaned with PBS, fixed with the Dako fluorescence mounting medium, and observed on Nikon Eclipse Ti2 microscope or Olympus BX51.

### Western blotting

Total cellular proteins were extracted with RIPA lysis buffer (Beyotime, China), which were then quantified by the bicinchoninic acid (BCA) kit (Beyotime, China). Proteins were separated by SDS PAGE and moved on a nitrocellulose membrane by electroblotting. Then, the membranes were blocked with TBS Containing 5% skim milk and 0.1%Tween20 for 2 h before the incubation with the diluted primary antibody at 4℃ overnoght. The primary antibodies used in this study were as follows: Bcl-2 (Cat#ab182858, Abcam), Bax (Cat# ab32503, Abcam), Bax (Cat# ab32503, Abcam), cleaved caspase3 (Cat#19677-1-AP, Proteintech), and GAPDH (Cat# ab8245, Abcam). Then the specific secondary antibodies were incubated at room temperature for 2 h. Each blot was exposed to film using the super signal chemiluminescence system (ECL, Pierce).

### Real-time quantitative PCR

Total RNA was extracted by the HiPure Total RNA Mini Kit (Magen, China). Real-time PCR was carried out using ChamQ TM Universal SYBR qPCR Master Mix. The sequences of primers are provided in Table 1.

**Table 1:** The primer sequences in the present study

Gene	Forward (5’–3’)	Reverse (5’–3’)
*COL2A1*	GAAGGATGGCTGCACGAAAC	CAACAATGGGAAGGCGTGAG
*ACAN*	ACATCCCAGAAAACTTCTTT	CGGCTTCGTCAGCAAAGCCA
*ADAMTS4*	GCTGTGCTATTGTGGAGGATGATGG	CCAGGGAAAGTCACAGGCAGATG
*MMP3*	CCTAGCGCTCTGATGTACCC	GGACTGGATGCCATTCACAT
*IL6*	ACCGGTCTTGTGGAGTTTCA	CAGGATCTTGGTACTCATGTGC
*TNF*	GAGCACTGAAAGCATGATCC	GAGAAGAGGCTGAGGCAGAA
*BCL2*	CATCCACCAAGAAGCTGAG	CCAACGGGAGAAGATGAAA
*BAX*	GAGAGGGAAATCGTGCGTGAC	CATCTGCTGGAAGGTGGACA
*CASP3*	TGGCCCTCTTGAACTGAAAG	TCCACTGTCTGCTTCAATACC
*GAPDH*	AGCCGCATCTTCTTGTGCAGTG	TGGTAACCAGGCGTCCGATACG

### Statistical analysis

All data analysis in this study was carried out with GraphPad Prism version 10.0.3. ANOVA was utilized for continuous variables involving three groups or more. Each experiment was performed in three replicates. Continuous variables will be presented as mean ± SD. The differences with *p* < 0.05 were considered statistically significant.

## Results

### Vitamin B6 blocks the progression of OA in CIA mice

**Fig. 1:**
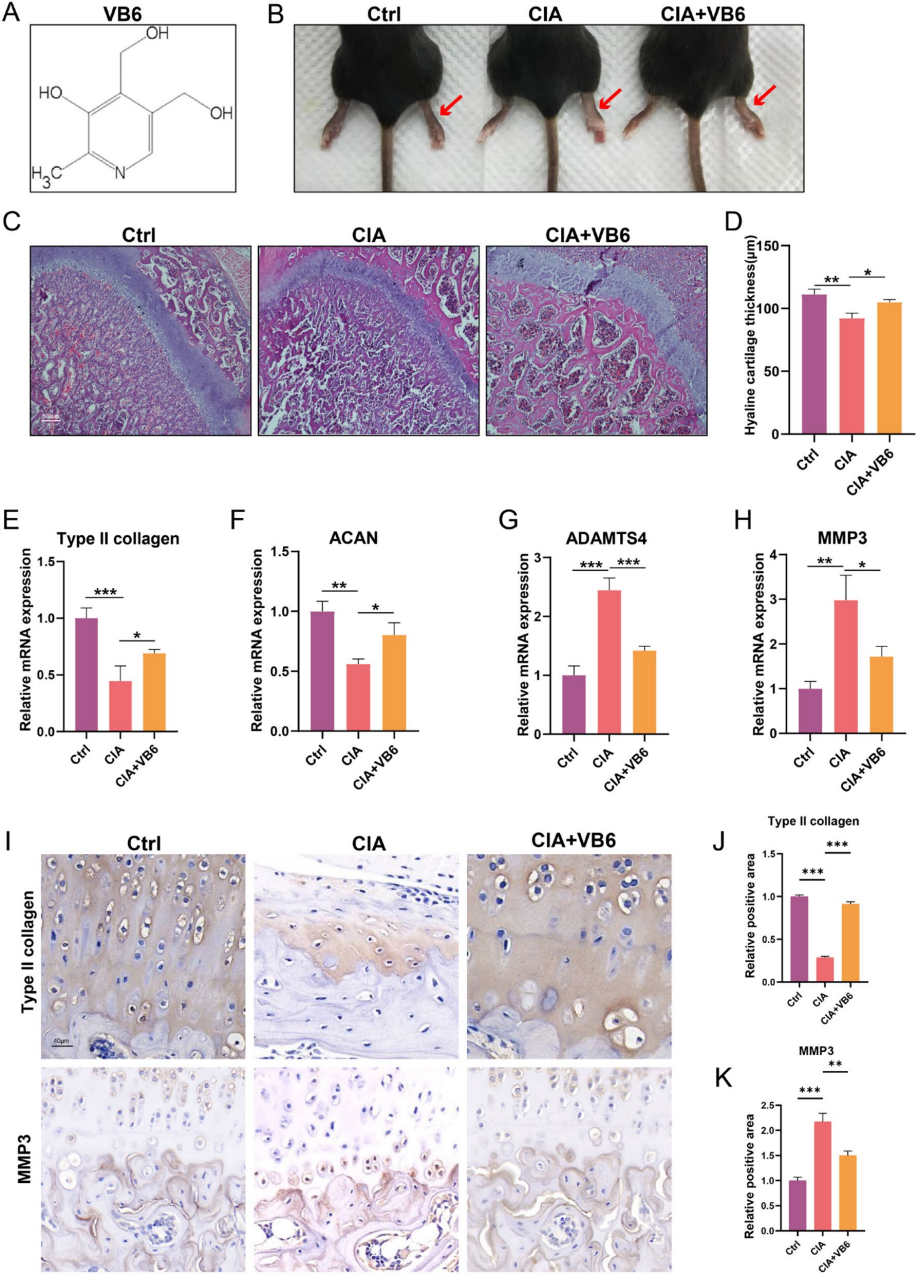
The effects of VB6 on CIA mice. (**A**) The chemical structure of the VB6. (**B**) The morphology of knees in mice. (**C**) The representative H&E staining results in groups. (**D**) The hyaline cartilage thickness of mice in various groups. (**E**-**H**) The mRNA levels of type II collagen, ACAN, ADAMTS4, and MMP3. (**I**) The representative immunohistochemical staining results of type II collagen and MMP3. (**J**-**K**) The semi-quantitative analysis results of immunohistochemical staining of type II collagen and MMP3. “*”, “**”, and “***” represent *P* < 0.05, *P* < 0.01, and *P* < 0.001, respectively

The chemical structure of the VB6 is displayed in Fig. 1A. Mice were randomly divised in three groups: control group, CIA group, and CIA + VB6 group. Mice in the CIA + VB6 group were treated with VB6 (40 mg/kg/day) once per day for 4 weeks before the CIA operation. The morphology of knees was filmed and the CIA group exhibited inflammatory morphogy, while the VB6 groups displayed a minimized inflamaton, confirming the anti-inflammatory of VB6 (Fig. 1B). Moreover, one week after the collagen-induced arthritis, hyaline cartilage thickness was measured using H&E to assess the potential impact of VB6 on chondrocytes alignment. H&E staining revealed reduced hyaline cartilage thickness and disordered chondrocytes alignment in the CIA group when compared with control group, indicating successful model construction **(**Fig. 1C, D**)**. The CIA + VB6 group exhibited significantly increased hyaline cartilage thickness than the CIA group **(**Fig. 1C, D**)**. In addition, the CIA + VB6 group exhibited distinguished alignment of chondrocytes and attenuated hypertrophy than the CIA group (Fig. 1C**).** What’s more, the mRNA levels of extracellular matrix metabolism (ECM)-related indicators were measured. The results revealed that both type II collagen and aggrecan (ACAN) were significantly higher in the CIA + VB6 group than those in the CIA group **(**Fig. 1E, F**)**, and that ADAMTS4 and MMP3 in the CIA + VB6 group were lower than those in the CIA group **(**Fig. 1G, H**)**, indicating that VB6 enhances the synthesis and suppress the degradation of ECM in CIA mice. The expression levels of MMP3 and type II collagen were assessed via immunohistochemical staining. The results showed that CIA treatment significantly reduced the positive area of type II collagen, which was remarkably restored by VB6 **(**Fig. 1I-J**).** In contrast, the positive area of MMP3 in the CIA group was significantly higher than that in the control group; compared to the CIA group, the CIA + VB6 group showed significantly lower positive area of MMP3 **(**Fig. 1I, K**)**.

### The VB6 cytotoxicity on chondrocytes

**Fig. 2:**
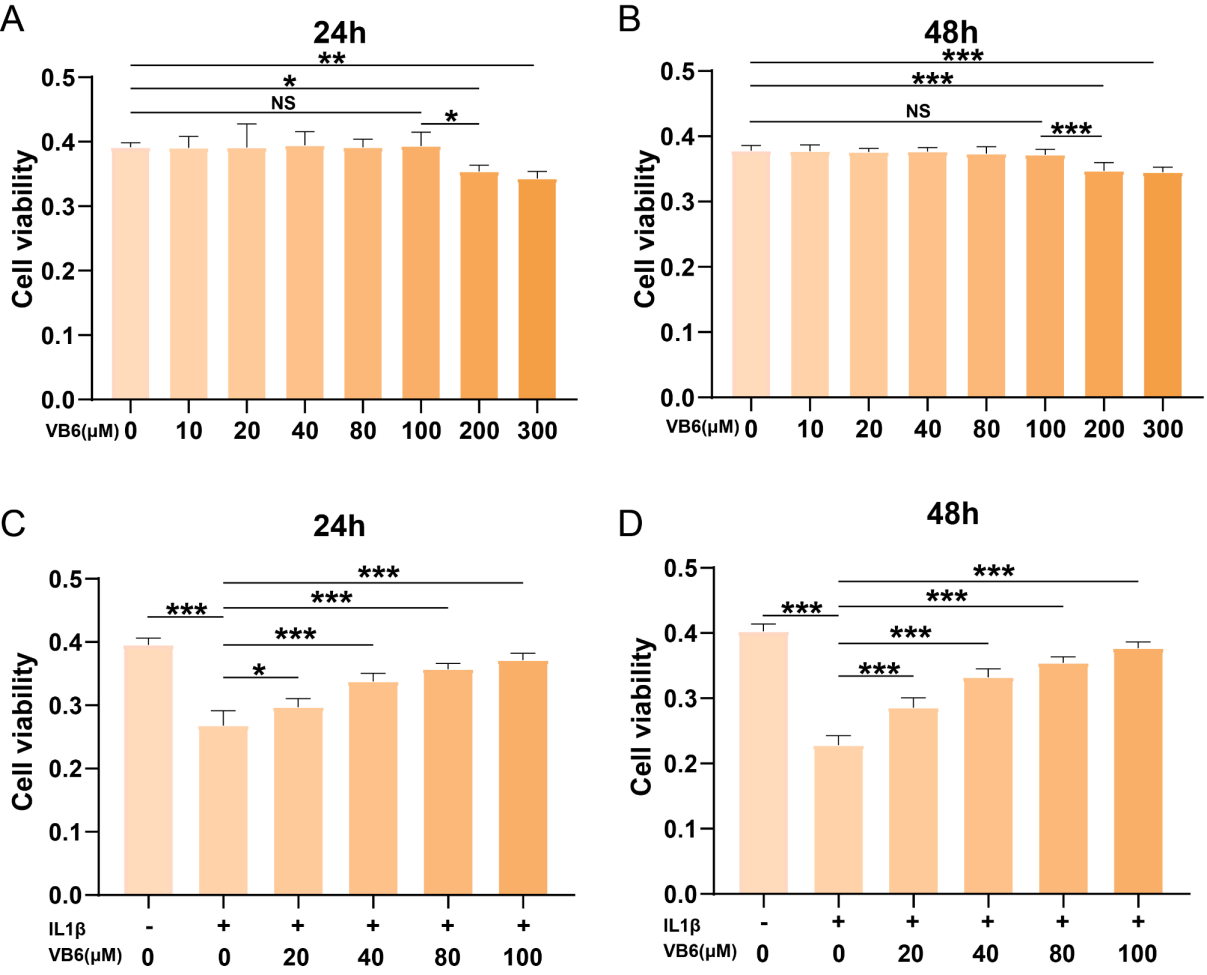
The cell viability of cells treated with various concentrations of VB6 and/or IL-1β. (**A**) The cell viability of chondrocytes treated with various concentrations of VB6 for 24 h. (**B**) The cell viability of chondrocytes treated with various concentrations of VB6 for 48 h. (**C**) The cell viability of chondrocytes treated with IL-1β and/or various concentrations of VB6 for 24 h. (**D**) The cell viability of chondrocytes treated with IL-1β and/or various concentrations of VB6 for 48 h. “*”, “**”, and “***” represent *P* < 0.05, *P* < 0.01, and *P* < 0.001, respectively

To evaluate the potential cytotoxicity of VB6 on chondrocytes, cells were cultivated for 24 and 48 h with VB6 at concentration gradient (0, 10, 20, 40, 80, 100, 200 and 300 µM). Then, through the CCK-8 assay we evaluated the cell viability. The results showed that VB6 displayed no significant toxicity on cell viability at concentrations that were not higher than 100 µM after 24–48 h of VB6 expsure (Fig. 2A, B). In addition, the cell survival rate of chondrocytes were significantly reduced in response to IL-1β stimulation (10 ng/ml), while VB6 at concentrations of 10, 50, or 100 µM strongly reversed the negative effects of IL-1β on chondrocytes (Fig. 2C, D). Therefore, VB6 at the concentrations of 10, 50, or 100 µM were selected for further experiments.

### Effect of VB6 on IL-1β-induced inflammation and ECM metabolism in chondrocytes

**Fig. 3:**
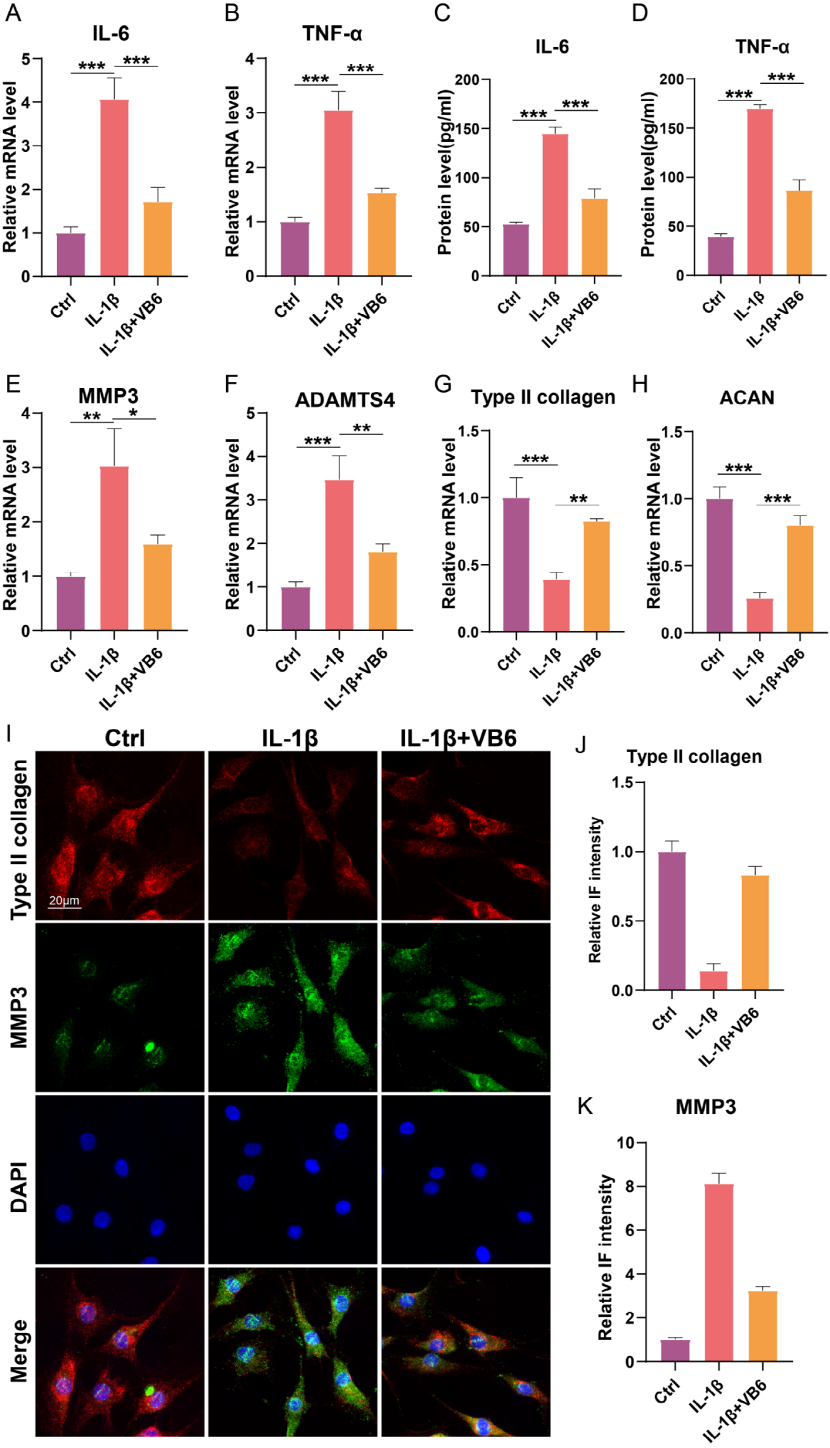
The role of VB6 in regulating inflammation and ECM metabolism in chondrocytes. (**A**-**B**) The mRNA levels of IL-6 and TNF-α. (**C**-**D**) The protein levels of IL-6 and TNF-α. (**E**-**H**)The mRNA levels of MMP3, ADAMTS4, type II collagen, and ACAN. (**I**) The immunofluorescence results of type II collagen and MMP3. (**J**-**K**) The semiquantitative analysis results of type II collagen and MMP3 immunofluorescence. “*”, “**”, and “***” represent *P* < 0.05, *P* < 0.01, and *P* < 0.001, respectively

To asses the effect of VB6 on inflammatory response, the mRNA levels and protein levels of IL-6 and TNF-α were detected. The results showed that the increased mRNA levels of both IL-6 and TNF-α induced by the treatment of IL-1β were significantly reduced by VB6 **(**Fig. 3A, B**)**; in addition, the ELISA results revealed that the protein levels of both IL-6 and TNF-α in the IL-1β + VB6 group were significantly lower than those in the IL-1β group **(**Fig. 3C, D**)**. The results demonstrated that VB6 could attenuate the inflammation induced by IL-1β in chondrocytes. To evaluate the role of VB6 in regulating ECM metabolism, both qRT-PCR and immunofluorescence were applied. The results showed that the mRNA levels of MMP3 and ADAMTS4 in the IL-1β + VB6 group were significantly lower than those in the IL-1β group (Fig. 3E, F); and the mRNA levels of both type II collagen and ACAN were remarkably higher than those in the IL-1β group (Fig. 3G, H). In addition, the immunofluorescence results showed that the immunofluorescence intensity of type II collagen which has been lowered by IL-1β treatment was significantly raised by VB6 (Fig. 3I, J), and the increased immunofluorescence intensity of MMP3 was significantly lowered by VB6 (Fig. 3I, K), indicating that VB6 prevents ECM degradation and promots anabolism.

### VB6 prevents apoptosis in chondrocytes

**Fig. 4:**
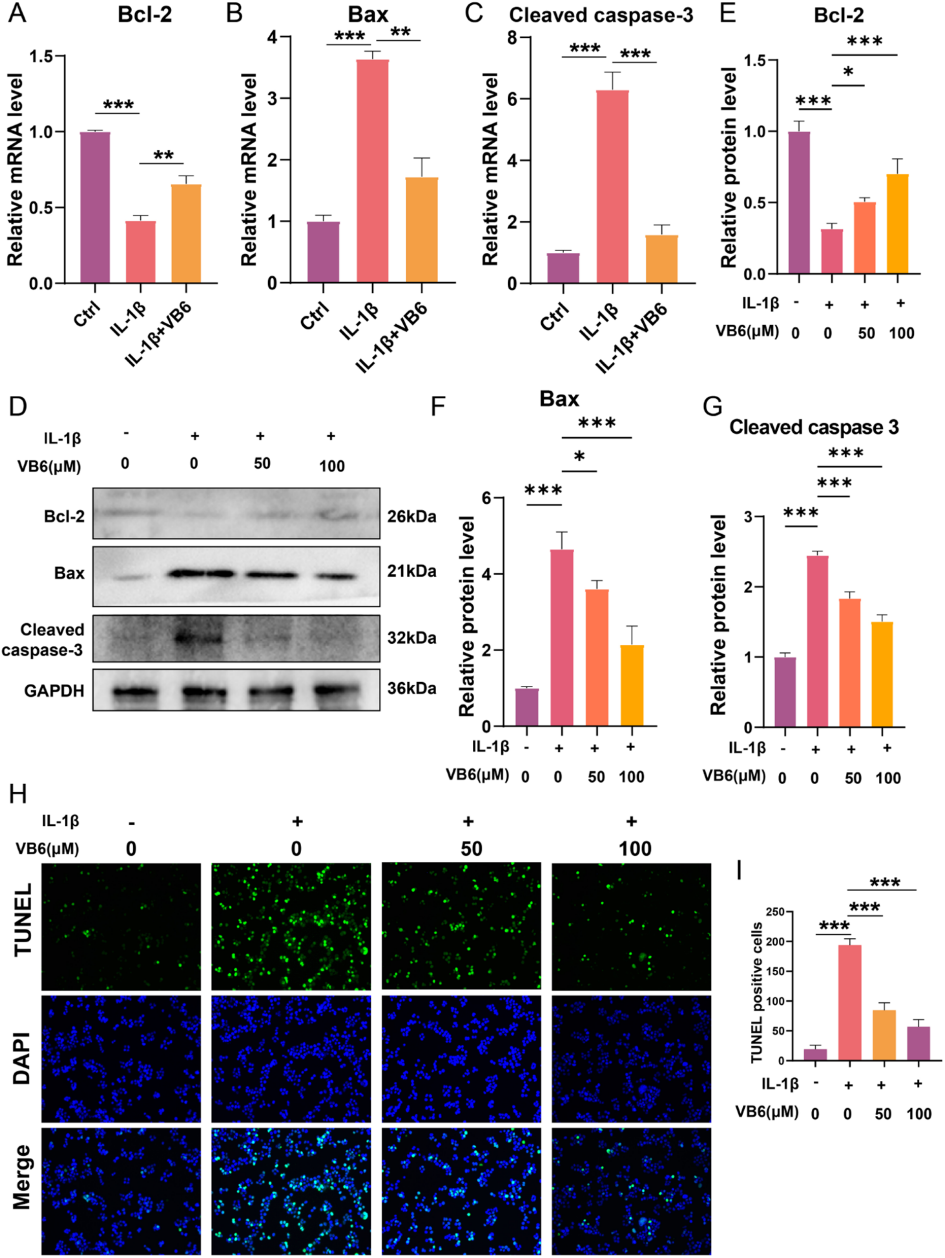
VB6 alleviates IL-1β-induced apoptosis in chondrocytes. (**A**-**C**) The mRNA levels of Bcl-2, Bax and cleaved caspase-3. (**D**) Western blot results of Bcl-2, Bax and cleaved caspase-3. (**E**-**G**) The semiquantitative analysis results of Bcl-2, Bax and cleaved caspase-3 blotting. (**H**) Tunel results of chondrocytes treated with IL-1β and/or VB6. (**I**) The quantitative analysis of Tunel positive cells. “*”, “**”, and “***” represent *P* < 0.05, *P* < 0.01, and *P* < 0.001, respectively

RT-qPCR results showed that the decreased mRNA level of anti-apoptotic Bcl-2, which is reduced by IL-1β, was reversed by VB6; in contrast, the increased mRNA levels of pro-apoptotic Bax and cleaved caspase-3 induced by IL-1β were reduced by VB6 treatment (Fig. 4A-C). Western blot analysis was applied to determine the expressions of Bax, cleaved caspase-3 and Bcl-2 in chondrocytes. The western blot results showed that the protein level of Bcl-2 was lowered after the IL-1β treatment, which was reversed by VB6 treatment **(**Fig. 4D, E). What’s more, IL-1β treatment dramatically increased the expressions of Bax, and cleaved caspase-3 compared to the control group, and this trend was reversed by VB6, suggesting that IL-1β might damage chondrocytes through apoptosis and VB6 could alleviate IL-1β-induced apoptosis (Fig. 4D-G). To verify the role of VB6 in regulating apoptosis in chondrocytes, TUNEL analysis was carried out. The results recealed that a significant amount of TUNEL positive cells was generated in chondrocytes treated by IL-1β (Fig. 4H, I). In contrast, chondrocytes that received VB6 supplements displayed a considerably low number of TUNEL positive cells when compared with those only treated by IL-1β(Fig. 4H, I), indicating that VB6 has anti-apoptotic effect on chondrocytes.

### Vitamin B6 exerts protective effects on knee joint tissue of mice

**Fig. 5:**
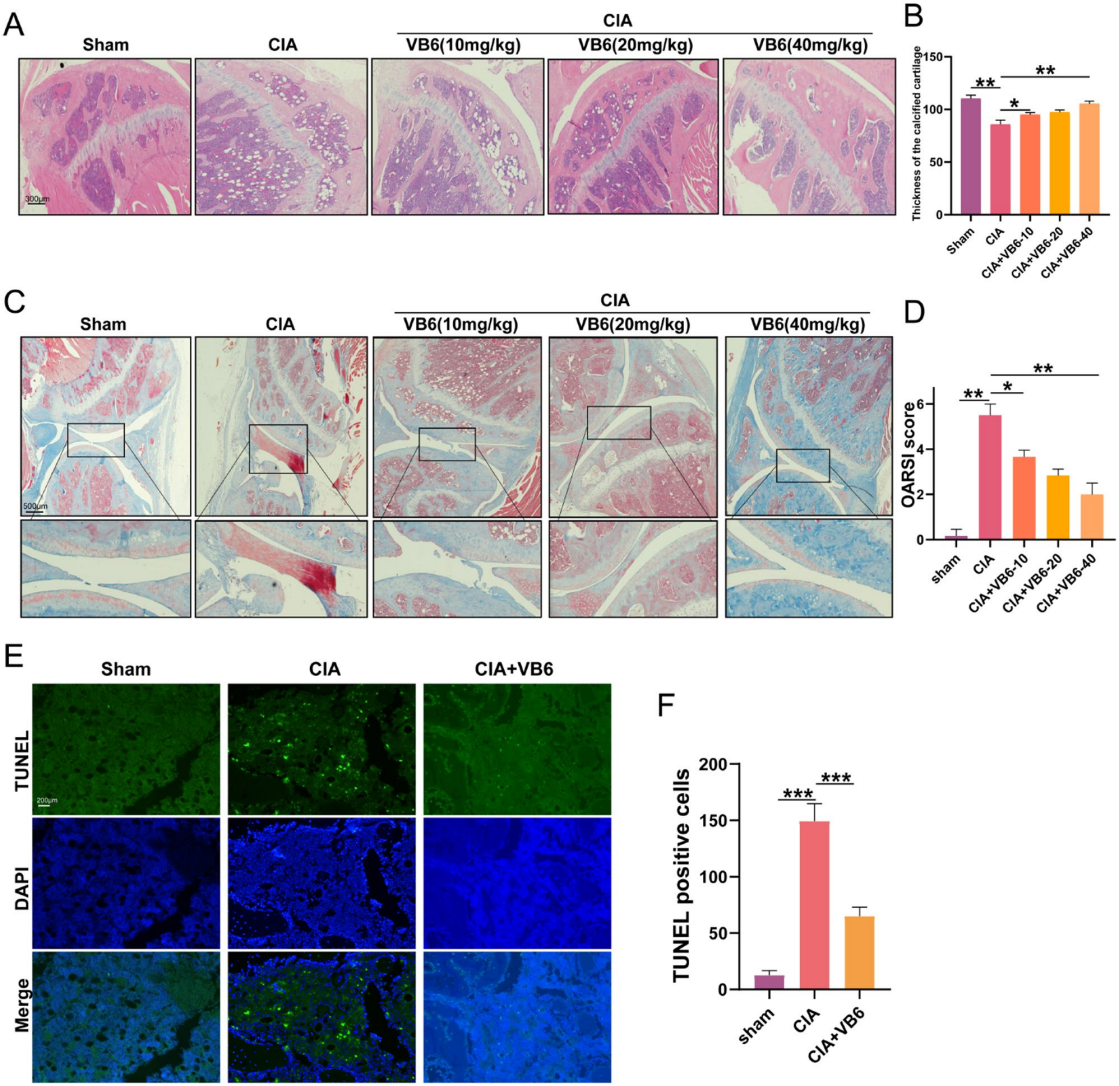
The effects of VB6 on the osteoarthritis in CIA mice. (**A**) The HE staining results of hyaline cartilage. (**B**) The hyaline cartilage thickness in CIA mice. (**C**) Masson staining of knee joint in CIA mice. (**D**) OARSI score of the mice. (**E**) Tunel results of the mice. (**F**) Tunel positive cells in mice. “*”, “**”, and “***” represent *P* < 0.05, *P* < 0.01, and *P* < 0.001, respectively

Finally, using mouse CIA model, we carried out an in vivo therapeutic assay to assess the benefits of VB6 in OA. 8 weeks following the operation, the medial of the tibia plateau from the operated hind limb was sectioned and examined. The HE staining showed that CIA mice treated with VB6 exhibited increased hyaline cartilage thickness compared to vehicle-treated CIA mice (Fig. 5A, B). In particular, the articular cartilage surface was rough and the Masson staining was low in the CIA group (Fig. 5C). The cartilage’s outermost layer in the CIA + VB6 group was smoother than those in the CIA group (Fig. 5C). In addition, the severity of OA in cartilage was determined using the Osteoarthritis Research Society International histology score approach (OARSI) score. The OARSI score displayed a lower level in the CIA + VB6 groups in comparison to that of the CIA group (Fig. 5C, D). The TUNEL tissue staining revealed a huge amount of cell dead in the articular cartilage surface of vehicle-treated CIA mice while CIA mice treated with VB6 displayed lower rate of cell dead (Fig. 5E, F). These results demonstrated that VB6 exerts chondroprotective effects on knee joint tissue of CIA mice.

## Discussion

Several studies have shown that IL-1β and tumor necrosis factor alpha (TNF-α) are the main pro-inflammatory cytokines involved in the pathophysiology of OA. Hence, the suppression mechasnism that generates IL-1β and TNF-α might constitute a potential protective path in articular cartilage [5]. In addition, the apoptotic rate of chondrocytes is highly enhanced in OA patients with severe cell survival decrease [20]. In this study we first confirmed VB6 exerts a protective role in regulating IL-1β-induced inflammation and apoptosis in OA.

Previous studies have demonstrated that VB6 played essential roles in various stress-responsive metabolisms, including protecting cartilage from degradation [21]. The levels of VB6 were found at the lowest concentration in patients with RA diseases compared with healthy controls [22, 23]. What’s more, it has been reported that ECM metabolism balance is essential for maintaining healthy articular cartilage tissues [24]. In our present study, the results showed that the ECM metabolism is improved by VB6 when compared with the control group. Expression levels of ECM-degrading enzymes including MMP3 and ADAMTS4 were remarkably decreased by VB6 in both the CIA mice and IL-1β-treated chondrocytes; whereas the levels of ECM markers including ACAN and type II collagen were raised after the VB6 treatment. Besides, inflammatory response also has been reported to be closely associated with the progression of ECM disorder related OA [25]. The findings in this present study revealed that VB6 could suppress the expression levels of inflammarory cytokines such as IL-6 and TNF-α. Our results demonstrated that VB6 attenuates osteoarthritis by improving the ECM metabolism and alleviating inflammation.

Apoptosis is one of the most important process in OA pathogenesis [26]. Herein, we have evidenced the role of VB6 in regulating apoptosis in CIA mice and IL-1β-treated chondrocytes by assesssing specific proteins including Bcl-2, Bax, and cleaved caspase-3 that are critical indicators mediating the apoptosis progression [27, 28]. In our present study, the results showed that VB6 exerts protective effects on CIA mice and attenuates apoptosis in both mice and chondrocytes. In addition, histopathological assessment demonstrates that VB6 treatment dramatically suppressed apoptosis in CIA mice. Our results is consistent with previous studies which showed that VB6 alleviates apoptosis in various diseases or disorders such as LPS-induced myocardial injury, high glucose-induced islet β cell death [29, 30]. A previous study reported that VB6-deficient rats developed advanced level of Hcy-induced atherosclerosis while supplementation in vitamin B6 significantly reversed the trend of Hcy-induced atherosclerosis [31]. Our findings indicated that oral administration of VB6 inhibits the progression of OA in CIA mice models. In addition, the decrease of thickness in the hyaline cartilage, alleviation of chondrocyte degradation and OARSI scores observed in CIA mice treated with VB6 indicated that VB6 could play a crucial role in OA therapy. In addition, in this present study, TUNEL showed that the supplementation of VB6 reduces the apoptotic rate of cells.

This study has several limitations. Although this study initially explored the role of VB6 in regulating osteoarthritis progression through alleviating apoptosis in mice, further mechanisms remain to be elucidated. And the assessment of OA could be more comprehensive in future study.

## Conclusions

In summary, findings in this present study demonstrated that sufficient dosages of VB6 induce considerable anti-apoptotic reactions in osteoarthritis. Additionally, the in vivo experiments demontstrated that CIA mice treated with VB6 exhibited a reduced progression of OA than CIA mice without VB6 treatment. Thus, this study revealed the protective role of VB6 in chondrocyte apoptosis and cartilage degeneration, and provided a new underlying mechanism and prospect for treating OA.

### Electronic supplementary material

Below is the link to the electronic supplementary material.

Supplementary Material 1

## Data Availability

The datasets used and/or analyzed during the current study are available from the corresponding author on reasonable request.

## Abbreviations

OA: Osteoarthritis
CIA: Collagen-Induced Arthritis
OARSI: Osteoarthritis Research Society International
ECM: Extracellular Matrix
VB6: Vitamin B6
TNF: Tumor Necrosis Factor
IL: Interleukin
OD: Absorbance Density
TUNEL: TdT-Mediated dUTP Nick End Labeling
BCA: Bicinchoninic Acid
