# Supplementary figures and images for: Vitamin B6 alleviates osteoarthritis by suppressing inflammation and apoptosis
Source: BMC Musculoskelet Disord. 2024 Jun 6;25:447. doi: 10.1186/s12891-024-07530-x (PMC11155127; doi:10.1186/s12891-024-07530-x)

BAX




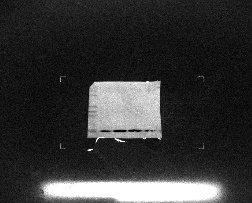




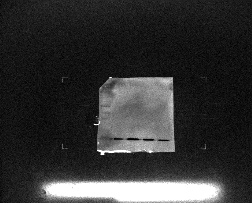




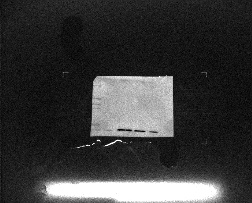


BCL2




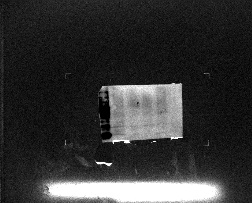




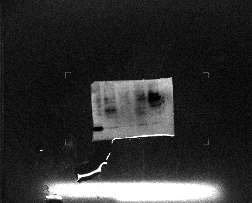




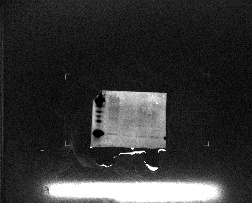


Cleaved caspase3




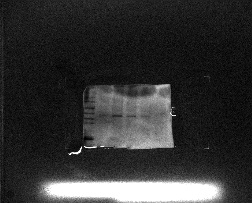




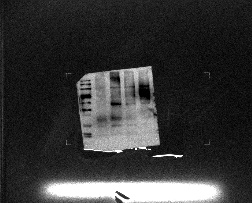




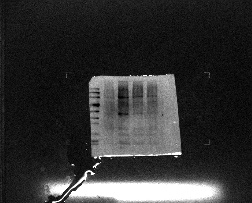


GAPDH




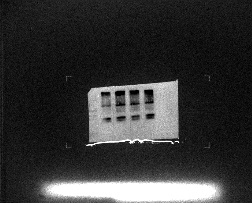




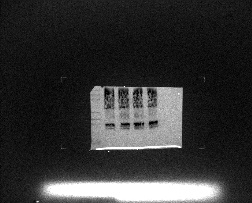




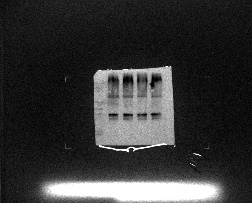

Supplement: Supplementary file 1 — Supplementary Material 1 [file 12891_2024_7530_MOESM1_ESM.docx]
